# Unemployment and ill health: a connection through inflammation?

**DOI:** 10.1186/1471-2458-9-410

**Published:** 2009-11-12

**Authors:** Jukka Hintikka, Soili M Lehto, Leo Niskanen, Anne Huotari, Karl-Heinz Herzig, Heli Koivumaa-Honkanen, Kirsi Honkalampi, Sanna Sinikallio, Heimo Viinamäki

**Affiliations:** 1 Department of Psychiatry, Kuopio University Hospital and Department of Clinical Medicine, University of Kuopio, FI-70210 Kuopio, Finland; 2 Department of Psychiatry, Kuopio University Hospital, Kuopio, Finland; 3 Department of Medicine, Kuopio University Hospital and Department of Clinical Medicine, University of Kuopio, Kuopio, Finland; 4 Institute of Biomedicine, Division of Physiology and Biocenter Oulu, University of Oulu, Oulu, Finland; 5 Department of Psychiatry, University of Oulu and Lapland Hospital District, Rovaniemi, Finland; 6 Department of Rehabilitation, Kuopio University Hospital, Kuopio, Finland

## Abstract

**Background:**

Unemployment is a source of acute and long-term psychosocial stress. Acute and chronic psychosocial stress can induce pronounced changes in human immune responses. In this study we tested our hypothesis that stress-induced low-grade tissue inflammation is more prevalent among the unemployed.

**Methods:**

We determined the inflammatory status of 225 general population subjects below the general retirement age (65 years in Finland). Those who had levels of both interleukin-6 (≥ 0.97 pg/mL) and high-sensitivity C-reactive protein (≥ 1.49 mg/L) above the median were assessed to have an elevated inflammatory status (n = 72).

**Results:**

An elevated inflammatory status was more common among the unemployed than among other study participants (59% versus 30%, p = 0.011). In the final multivariate model, those who were unemployed had over five-fold greater odds for having an elevated inflammatory status (OR 5.20, 95% CI 1.55-17.43, p = 0.008).

**Conclusion:**

This preliminary finding suggests that stress-induced low-grade inflammation might be a link between unemployment and ill health.

## Background

In June 2009, approximately 45 million (8.3%) people living in the OECD countries were unemployed, and the figures were rapidly increasing due to the severe economic recession [1]. In some underdeveloped countries, estimated unemployment rates may be as high as 80 to 90% [2].

Ill health is common among the unemployed, and the causal relationship may be bidirectional. Poor health may lead to unemployment or be an obstacle to employment opportunities, while, vice versa, being unemployed may have an adverse effect on one's health [3,4]. Several health problems such as stress symptoms, mental disorders, hypertension, and coronary heart disease are associated with unemployment [5-8]. However, unemployment periods may protect workers from musculoskeletal diseases [9]. Many socio-demographic factors such as sex, age, education, alcohol consumption, obesity, and other adverse life-styles modify the relationship between unemployment and health [7], and even unemployment and mortality [10].

During recent years, studies have suggested that acute and chronic psychosocial stress can induce pronounced changes in human immune responses and that these changes are predominantly mediated via neuroendocrine mediators [11-13]. Furthermore, high levels of inflammation seem to associate with several somatic diseases, depression, and disease progression [14-16]. These associations suggest that high levels of inflammation might be a mediator between unemployment and ill health. Nevertheless, we found only two previous studies suggesting that some aspects of immune function may be altered following the loss of work [17,18].

Arnetz et al. found significant decreases in phytohemagglutinin reactivity of lymphocytes and in reactivity to a purified protein derivative of tuberculin in unemployed women after nine months [17]. Janicki-Deverts et al. reported that discrete episodes of unemployment may have long-term implications for future CRP level in young males [18].

High levels of proinflammatory cytokine, interleukin-6 (IL-6), and high-sensitivity C-reactive protein (hs-CRP) are indicators of inflammation. Yaffe et al. originally suggested, and Capuron et al. repeated the suggestion that medians of IL-6 and hs-CRP could be used together as indicators of low-grade tissue inflammation [14,19]. In the current study using a sample of the Finnish general population we applied this suggestion to test our hypothesis - formulated on the basis of current evidence - that stress-induced low-grade tissue inflammation is more prevalent among the unemployed.

## Methods

As a part of an ongoing follow-up study on a sample of the Finnish general population we determined the inflammatory status in 225 individuals below the general retirement age (65 years in Finland). There were 93 men and 132 women in the sample. The mean age was 52.6 (SD 8.2, range: 32 - 64) years in men and 52.0 (SD 7.6, range 32-64) years in women. The study period was from October 2004 to April 2006. Approval to conduct the study was obtained from the Ethics Committee of Kuopio University Hospital and the University of Kuopio. All procedures were carried out with the adequate understanding of the study participants, who gave written informed consent before entering the study.

The respondents completed a standardized questionnaire that screened sociodemographic variables (sex, age, marital status, length of education). They were also asked how often they had used alcohol over the past twelve months (classified as less/more than twice a week), whether they were current daily smokers and whether they had physical diseases diagnosed by a doctor. The participants were also asked whether there had been subjective economic hardship during the previous twelve months ("How is your current financial situation?" (good, fairly good = good financial situation, fairly poor, poor = economic hardship). The questionnaire also included the 21-item Beck Depression Inventory, which was used to indicate the level of depressive symptoms [20].

In addition, the employment status was inquired and those who reported being unemployed (n = 19, 8.4% of the study sample) were compared with the others in final analyses. The frequency of unemployment in the sample was at the same level as the total unemployment rate in Finland during the study period (7.7% in December 2004 and 7.6% in December 2005) [21]. Of the other study participants, 131 (63.6%) were at work, 14 (6.8%) on sick leave, 52 (25.2%) had retired, 3 (1.5%) were students and 6 (2.9%) were voluntarily not at work. Of all study participants, 25 (11.1%) had been unemployed in 1998, 31 (13.8%) in 1999 and 28 (12.4%) in 2001.

Height and body weight were measured in light clothing without shoes, and the body mass index (BMI; kg/m^2^) was estimated. The laboratory measurements were carried out in the medical laboratory of Kuopio University Hospital. The participants came for venous blood sampling at 8 am, after having been instructed to fast for the previous 12 hours.

The measurement of serum high-sensitivity C-reactive protein was carried out according to a routine protocol using a kinetic immunoturbidimetric method (Beckman Coulter CRH High Sensitivity Protein Reagent, Beckman Coulter CAL 5 Plus) and analyzed using an IMMAGE Immunochemistry System (Beckman Coulter, USA).

For the cytokine analyses, the venous blood samples were stored at -80°C until run. The levels of IL-6 (pg/mL) were analyzed by multiplexing with Bio-Plex Human Cytokine Panel 1 utilizing a Bio-Plex instrument based on Luminex xMAP technology (Bio-Rad Laboratories Inc., CA, US). Before analyses, the samples were centrifuged for 15 min at 3000 rpm, and diluted 1:2 in an appropriate sample matrix. The assays were performed according to the manufacturer's instructions. The intra- and interassay variations for the IL-6 analysis were 4.6-13.8% and 3.7-17.2%, respectively.

As originally suggested by Yaffe et al. and later by Capuron et al., we used a proinflammatory cytokine, interleukin-6 (IL-6), and high-sensitivity C-reactive protein (hs-CRP) as indicators of low-grade inflammation [14,19]. In this study, levels above the median for both IL-6 (≥ 0.97 pg/mL) and hs-CRP (≥ 1.49 mg/L) indicated an elevated inflammatory status (n = 72).

In univariate analyses we used the χ^2 ^test for categorical variables, Student's t-test for normally distributed continuous variables and Mann-Whitney U-test for continuous variables with a skewed distribution. Pearson's correlation coefficients (r) were calculated between IL-6, hs-CRP, age, length of education, BDI and BMI. Linear regression models were tested separately for IL-6 and hs-CRP. Inflammatory markers were log-transformed for these analyses. Finally, several forced multivariate logistic regression models were calculated to test whether there were independent associations between being or having been unemployed, and having a high inflammatory status. The models were adjusted for sex, age, marital status, economic hardship, education, smoking, alcohol consumption, some common somatic diseases, the Beck Depression Inventory score, and the body mass index.

## Results

Basic characteristics of the sample are presented in Table 1. The only statistically significant difference between the unemployed and other study participants was in the frequency of economic hardship (p = 0.012).

**Table 1:** Basic characteristics of the sample

	The unemployed (n = 19)	Other study participants (n = 206)
Men (%)	42	41
Age (years; mean (SD))	49.2 (9.1)	52.6 (7.7)
Marital status: single (%)	21	17
Economic hardship (%) *	42	18
Daily smoking (%)	26	23
Alcohol use 2×/week or more (%)	26	17
Education (years; mean (SD))	11.5 (2.7)	12.3 (3.5)
Hypertension (%)	32	30
Coronary heart disease (%)	4	11
Musculoskeletal symptoms (%)	11	20
BDI total score (mean (SD))	10.0 (8.5)	8.1 (8.4)
BMI (kg/m^2^; mean (SD))	28.0 (5.8)	27.2 (5.4)
IL-6 (pg/mL; mean (SD))	27.51 (101.32)	3.21 (9.53)
hs-CRP (mg/L; mean (SD))	3.56 (4.21)	2.74 (3.85)

SD = standard deviation, BDI = Beck Depression Inventory; BMI = body mass index. IL-6 = interleukin-6. hs-CRP = high-sensitivity C-reactive protein.

* χ^2 ^= 6.34, df = 1, p = 0.012.

There was a trend that the unemployed had higher IL-6 levels than other study participants (p = 0.067). No other differences were found in levels of IL-6 or hs-CRP in relation to gender, economic situation, smoking, alcohol use and somatic diseases (data not shown).

Il-6 and hs-CRP correlated significantly with each other (r = 0.271, p < 0.01). Moreover, IL-6 correlated positively with BMI (r = 0.154, p < 0.05) and negatively with length of education (r = -0.173, p < 0.01), and hs-CRP correlated positively with BMI (r = 0.439, p < 0.01) and the BDI score (r = 0.131, p < 0.05). No other statistically significant correlations were found between hs-CRP or IL-6 and age, BMI, BDI score or length of education.

Unemployment associated independently with IL-6 levels in a sex- and age-adjusted linear regression model (β = 0.145, p = 0.03). The association remained statistically significant when the model was separately adjusted for sex, age, marital status, economic hardship, smoking, alcohol use, education, somatic diseases, BDI total score and BMI (p = 0.22 - 0.46). However, after full adjustment there was only a trend for an independent association between unemployment and IL-6 (p = 0.075). Respective associations between unemployment and hs-CRP were not statistically significant.

An elevated inflammatory status (both IL-6 and hs-CRP above the median) was more common among unemployed study participants than the others (11/19, 58% versus 61/206, 30%, χ^2 ^= 6.40, df = 1, p = 0.01). Moreover, those who had an elevated inflammatory status had fewer years of education (11.5 (SD 3.6) versus 12.5 (SD 3.3) years, t = 1.96, df = 220, p = 0.05) and a higher body mass index (29.4 (SD 6.9) versus 26.3 (SD 4.2), Mann-Whitney U = 4041.00, Z = -2.96, p = 0.003) than the others.

In the final logistic regression model, which was adjusted for sex, age, marital status, economic hardship, smoking, alcohol use, education, somatic diseases, BDI total score and BMI, those who were unemployed had significantly increased odds for having an elevated inflammatory status (odds ratio (OR) 5.20, 95% confidence interval (CI) 1.55-17.43, p = 0.008) (Table 2). A higher body mass index was the only other variable which also associated statistically significantly (p < 0.001) with inflammatory status.

**Table 2:** Odds for having an elevated inflammatory status

	Crude OR (95% CI)	Adjusted OR (95% CI)
Sex (men/women)	0.70 (0.40-1.24)	0.69 (0.37-1.32)
Age (years)	1.02 (0.98-1.06)	1.04 (0.99-1.09)
Marital status (other/single)	0.81 (0.38-1.06)	0.70 (0.29-1.72)
Economic hardship (no/yes)	0.95 (0.47-1.92)	0.57 (0.21-1.51)
Daily smoking (no/yes)	1.73 (0.92-3.28)	2.12 (0.99-4.57)
Alcohol use (less/more than 2×/week)	1.08 (0.52-2.24)	0.90 (0.40-2.06)
Education (years)	0.92 (0.84-1.00)	0.99 (0.89-1.10)
Hypertension (no/yes)	1.64 (0.90-2.98)	1.29 (0.62-2.69)
Coronary heart disease (no/yes)	0.20 (0.03-1.60)	0.15 (0.02-1.33)
Musculoskeletal symptoms (no/yes)	0.87 (0.42-1.78)	0.91 (0.38-2.16)
BDI total score	1.01 (0.98-1.05)	1.00 (0.96-1.05)
BMI (kg/m2)	1.12 (1.06-1.18)	1.13 (1.06-1.20)
Unemployed (no/yes)	2.84 (1.10-7.33)	5.20 (1.55-17.43)

OR = odds ratio; CI = confidence interval; BDI = Beck Depression Inventory; BMI = body mass index.

We also tested whether being unemployed in 1998, 1999 or 2001 was associated with the inflammatory status in 2005. Unemployment in none of these years reached statistical significance when added to the model presented in Table 2. The unemployed had the following adjusted odds for an elevated inflammatory status: OR 2.67 (95% CI 0.87-8.18), p = 0.086 in 1998, OR 1.52 (95% CI 0.57-4.07), p = 0.40 in 1998 and OR 0.79 (95% CI 0.27 - 2.30), p = 0.67 in 2001. The association between being unemployed and having an elevated inflammatory status in 2005 remained statistically significant after adjustments for unemployment in 1998, 1999 and 2001. The odds were 4.12 (95% CI 1.17-14.58), p = 0.028 in 1998, 3.94 (95% 1.13-13.77), p = 0.032 in 1999 and 5.33 (95% CI 1.45-19.62), p = 0.012 in 2001.

Because very high levels of IL-6 and hs-CRP may indicate acute infection, we also tested a fully adjusted model where participants who had very high (mean + 3 SD) levels of IL-6 (> 97.70 pg/mL) and hs-CRP (> 14.45 mg/L) had been excluded. The independent association between unemployment and low-grade tissue inflammation remained essentially the same as in our final model using the whole study population (OR 4.91, 95% CI 1.42 - 16.98).

## Discussion

Unemployment and an elevated inflammatory status indeed appeared to be associated, as we hypothesized. The association remained significant even after adjustments for several possible confounders.

Although preliminary and certainly in a need of replication, these findings suggest that unemployment is among those psychosocial stressors that may affect the human immune system. Furthermore, since disturbances in the immune system are associated with an increased risk of several illnesses, stress-induced low-grade inflammation might be among the links between unemployment and ill health.

Being unemployed seven, six or four years earlier did not associate with an elevated inflammatory status in 2005 when adjusted for unemployment in 2005. This contradicts the findings from previous studies in which periods of unemployment have associated with disturbed immune function even some years later [17,18]. However, different measures of immune function may give different findings concerning its association with unemployment.

Previously, Janicki-Deverts et al. have reported that discrete episodes of unemployment and higher CRP levels are associated [18]. We did not find such an association. Instead, levels of IL-6 seemed to associate with unemployment even after several adjustments. However, an inflammatory marker that was based on above median levels of both IL-6 and hs-CRP was the best indicator of a connection between unemployment and low-grade tissue inflammation. The same method has been used to indicate that inflammation is one determinant of depressive symptoms in individuals with metabolic syndrome and that metabolic syndrome contributes to cognitive impairment in elderly people with a high level of inflammation [14,19].

Based on the evidence, the American Heart Association (AHA) and the Center for Disease Control (CDC) have recommended cut-offs of 1.0 and 3.0 mg/L for hs-CRP to be used for categorization into a low, intermediate, and high risk of cardiovascular diseases [22]. The median (1.49 mg/L) used as a cut-off level in this study indicates intermediate risk. IL-6 does not have any established cut-off level for clinically significant inflammation. Thus, although the same methodology has been used as a measure of inflammation in several studies, its clinical significance has not yet been fully established.

The small sample size is a major shortcoming of this study. Another limitation is that we did not know how long the study participants had been unemployed. It is not, however, self-evident that negative consequences on health are more probable if one has been unemployed for a long time [4]. Moreover, our cross-sectional study design does not allow us to exclude the possibility that study participants had had an elevated inflammatory status prior to unemployment. However, the final multivariate model, which suggested an independent association between unemployment and inflammation, was adjusted for several confounding variables, which decreases the risk of misinterpretation. Finally, dichotomization of continuous variables may have increased the risk of spurious findings in multivariate models [23].

We used the Luminex method in cytokine analyses. Luminex measurements of unstimulated plasma IL-6 levels may be different from those obtained with traditional enzyme-linked immunosorbant assays (ELISA), the gold standard of current peptide immunoassays. This may make comparisons between studies difficult. However, the correlation between the Luminex method and other multiplex assays has been shown to be sufficient, and the correlations between multiplex analyses and traditional ELISA techniques have also been demonstrated to be high in several papers [24-28]. In our study, regardless of the observed overall levels of IL-6 and hs-CRP, an elevated inflammatory status associated independently with unemployment. Finally, a limitation of the current study is that the samples were not assayed in a duplicate. However, the measurement principle of the Luminex assay method could overrule this limitation, at least partially, as the values obtained for the measured analyte were average values based on 100 bead measurements.

## Conclusion

We found that unemployment is associated with low-grade tissue inflammation, which may be a stress-induced link between unemployment and ill health. The finding, however, is preliminary and needs replication to be confirmed. Further studies are therefore warranted.

## Competing interests

The authors declare that they have no competing interests.

## Authors' contributions

JH performed the statistical analysis and drafted the manuscript. SML, LN, H-KH, KH and SS participated in acquisition of data and helped to draft the manuscript. AH and K-HH carried out the immunoassays and helped to draft the manuscript. HV participated in the design of the study and helped to draft the manuscript. All authors read and approved the final manuscript.

## Pre-publication history

The pre-publication history for this paper can be accessed here:

http://www.biomedcentral.com/1471-2458/9/410/prepub

## References

[B1] OECD StatExtracts http://stats.oecd.org/WBOS/Index.aspx?QueryName=251&QueryType=View

[B2] CIA - The World Factbook https://www.cia.gov/library/publications/the-world-factbook/rankorder/2129rank.html

[B3] Bartley M Unemployment and ill health: understanding the relationship J Epidemiol Community Health 1994 48 333 337 10.1136/jech.48.4.333 7964329 PMC1059979

[B4] Böckerman P Ilmakunnas P Unemployment and self-assessed health: evidence from panel data Health Economics 2009 18 161 179 10.1002/hec.1361 18536002

[B5] Cook DG Cummins RO Bartley MJ Shaper AG Health of unemployed middle-aged men in Great Britain Lancet 1982 319 8284 1290 1294 10.1016/S0140-6736(82)92852-5 6123028

[B6] Hammarström A Health consequences of youth unemployment Public Health 1994 108 403 412 10.1016/S0033-3506(94)80097-9 7997489

[B7] Leino-Arjas P Liira J Mutanen P Malmivaara A Matikainen E Predictors and consequences of unemployment among construction workers: prospective cohort study BMJ 1999 319 600 605 10473472 10.1136/bmj.319.7210.600 PMC28210

[B8] Breslin FC Mustard C Factors influencing the impact of unemployment on mental health among young and older adults in a longitudinal, population-based survey Scand J Work Environ Health 2003 29 5 14 12630430 10.5271/sjweh.698

[B9] Heponiemi T Elovainio M Manderbacka K Aalto A-M Kivimäki M Keskimäki I Relationship between unemployment and health among health care professionals: health selection or health effect? J Psychosom Res 2007 63 425 431 10.1016/j.jpsychores.2007.04.005 17905052

[B10] Lundin A Lundberg I Hallsten L Ottosson J Hemingsson T Unemployment and mortality - a longitudinal prospective study on selection and causation in 49 321 Swedish middle aged men J Epidemiol Community Health 2009 10.1136/jech.2008.079269 19289388

[B11] Kemeny ME Schedlowski M Understanding the interaction between psychosocial stress and immune-related diseases: a stepwise progression Brain Behav Immunity 2007 21 1009 1018 10.1016/j.bbi.2007.07.010 17889502

[B12] Sorrells SF Sapolsky RM An inflammatory review of glucocorticoid actions in the CNS Brain, Behav Immunity 2007 21 259 272 10.1016/j.bbi.2006.11.006 PMC1997278 17194565

[B13] Leonard BE Myint A The psychoneuroimmunology of depression Hum Psychopharmacol 2009 24 165 175 19212943 10.1002/hup.1011

[B14] Capuron L Su S Miller AH Bremner JD Goldberg J Vogt GJ Depressive symptoms and metabolic syndrome: is inflammation the underlying link? Biol Psychiatry 2008 64 896 900 10.1016/j.biopsych.2008.05.019 18597739 PMC2621309

[B15] Picciotto S Forastiere F Pistelli R Koenig W Lanki T Ljungman P Determinants of plasma interleukin-6 levels among survivors of myocardial infarction Eur J Cardiovasc Prev Rehabil 2008 15 631 638 10.1097/HJR.0b013e3283069d9a 19177625

[B16] Miller AH Maletic V Raison CL Inflammation and its discontents: the role of cytokines in the pathophysiology of major depression Biol Psychiatry 2009 65 732 741 10.1016/j.biopsych.2008.11.029 19150053 PMC2680424

[B17] Arnetz BB Wasserman J Petrini B Brenner S-O Levi L Eneroth P Immune function in unemployed women Psychosom Med 1987 49 3 12 3823348 10.1097/00006842-198701000-00001

[B18] Janicki-Deverts D Cohen S Matthews KA Cullen MR History of unemployment predicts future elevations in C-reactive protein among male participants in the Coronary Artery Risk Development in Young Adults (CARDIA) study Ann Behav Med 2008 36 176 185 10.1007/s12160-008-9056-5 18784972

[B19] Yaffe K Kanaya A Lindquist K Simonsick EM Harris T Shorr RI The metabolic syndrome, inflammation, and risk of cognitive decline JAMA 2004 292 2237 2242 10.1001/jama.292.18.2237 15536110

[B20] Beck AT Ward CH Mendelson M Mock J Erbaugh J An inventory for measuring depression Arch Gen Psychiatry 1961 4 561 571 13688369 10.1001/archpsyc.1961.01710120031004

[B21] Statistics Finland http://www.stat.fi/ajk/tiedotteet/v2006/tiedote_006_2006-01-24_en.html

[B22] Pearson TA Mensah GA Alexander RW Anderson JL Cannon RO III Criqui M Fadl YY Fortmann SP Hong Y Myers GL Rifai N Smith SC Jr Taubert K Tracy RP Vinicor F Markers of inflammation and cardiovascular disease: application to clinical and public health practice: a statement for healthcare professionals from the Centers for Disease Control and prevention and the American Heart Association Circulation 2003 107 499 511 10.1161/01.CIR.0000052939.59093.45 12551878

[B23] Babyak MA What you see may not be what you get: a brief, nontechnical introduction to overfitting in regression-type models Psychosom Med 2004 66 411 421 10.1097/01.psy.0000127692.23278.a9 15184705

[B24] Khan SS Smith MS Reda D Suffredini AF McCoy JP Jr Multiplex bead array assays for detection of soluble cytokines: comparisons of sensitivity and quantitative values among kits from multiple manufacturers Cytometry B Clin Cytom 2004 61 35 39 10.1002/cyto.b.20021 15351980

[B25] Khan IH Krishnan VV Ziman M Janatpour K Wun T Luciw PA Tuscano J A comparison of multiplex suspension array large-panel kits for profiling cytokines and chemokines in rheumatoid arthritis patients Cytometry B Clin Cytom 2009 76 159 168 10.1002/cyto.b.20452 PMC4425447 18823005

[B26] de Jager W te Velthuis H Prakken BJ Kuis W Rijkers GT Simultaneous detection of 15 human cytokines in a single sample of stimulated peripheral blood mononuclear cells Clin Diagn Lab Immunol 2003 10 133 139 12522051 10.1128/CDLI.10.1.133-139.2003 PMC145264

[B27] Elshal MF McCoy JP Multiplex bead array assays: performance evaluation and comparison of sensitivity to ELISA Methods 2006 38 317 323 10.1016/j.ymeth.2005.11.010 16481199 PMC1534009

[B28] Leng SX McElhaney JE Walston JD Xie D Fedarko NS Kuchel GA ELISA and multiplex technologies for cytokine measurement in inflammation and aging research J Gerontol A Biol Sci Med Sci 2008 63 879 884 18772478 10.1093/gerona/63.8.879 PMC2562869

